# Health behaviors and risk factors in those who use complementary and alternative medicine

**DOI:** 10.1186/1471-2458-7-217

**Published:** 2007-08-27

**Authors:** Richard L Nahin, James M Dahlhamer, Beth L Taylor, Patricia M Barnes, Barbara J Stussman, Catherine M Simile, Marc R Blackman, Margaret A Chesney, Morgan Jackson, Heather Miller, Kim K McFann

**Affiliations:** 1 National Center for Complementary and Alternative Medicine, National Institutes of Health, 9000 Rockville Pike, Bethesda, MD 20892-2182, USA; 2 National Center for Health Statistics, Division of Health Interview Statistics Centers for Disease Control and Prevention, 3311 Toledo Road, Hyattsville, MD 20782, USA; 3 Barbara Davis Center for Childhood Diabetes, University of Colorado at Denver Health Sciences Center, Aurora, CO 80045-6511, USA

## Abstract

**Background:**

Surveys have generally found that individuals more likely to use complementary and alternative medicine are female, live in the western United States, are likely to have a health complaint, and have a higher socioeconomic status than do nonusers. What is not known is the extent to which those who use complementary and alternative medicine also engage in positive health behaviors, such as smoking cessation or increased physical activity and/or exhibit fewer health risk factors such as obesity. This has been identified as a key research question in a recent Institute of Medicine report. In the present study we sought to determine whether the use of complementary and alternative medicine is associated with health behaviors or risk factors known to impact on health status.

**Methods:**

The current study is a cross-sectional regression analysis using data from the 2002 National Health Interview Survey. Data were collected in-person from 31,044 adults throughout the 50 states and the District of Columbia.

**Results:**

After controlling for a range of other factors, we found that engaging in leisure-time physical activity, having consumed alcohol in one's life but not being a current heavy drinker, and being a former smoker are independently associated with the use of CAM. Obese individuals are slightly less likely to use CAM than individuals with a healthy body-mass index. No significant associations were observed between receipt of an influenza vaccine and CAM use.

**Conclusion:**

Those engaging in positive health behaviors and exhibiting fewer health risk factors are more likely to use CAM than those who forgo positive health behaviors or exhibit more health risk factors. The fact that users of CAM tend to pursue generally healthy lifestyles suggests that they may be open to additional recommendations toward optimizing their health.

## Background

Complementary and alternative medicine (CAM) comprises a diverse set of healing philosophies, therapies and products. Increasingly, people with chronic health conditions are turning toward CAM for relief [1,2]. Prior national surveys on CAM use in the United States (U.S.) have focused on sociodemographic factors such as gender, region of residence, income, and education [1-3]. These surveys have generally found that individuals more likely to use CAM are female, live in the western U.S., are likely to have a health complaint, and have a higher socioeconomic status than do nonusers. What is not known is the extent to which those who use CAM also engage in positive health behaviors, such as smoking cessation or increased physical activity and/or exhibit fewer health risk factors such as obesity, and whether these behaviors are independent of health status and other factors associated with CAM use. These have been identified as key research questions in a recent Institute of Medicine report on CAM [4].

Some individuals who practice a range of positive health behaviors and place a greater value on wellness and disease prevention may judge they are better served by CAM than by the conventional medical system [5,6]. Furthermore, it is known that individuals who use CAM are more likely to take an active role in maintaining their health [7-9]. Therefore, we hypothesized that individuals who engage in positive health behaviors and/or exhibit fewer health risk factors are more likely to use CAM than those who forgo positive health behaviors or exhibit more health risk factors, and that these associations are independent of current health status, healthcare access and utilization, and sociodemographic factors. To test this hypothesis, we perform logistic regression analysis on data from the 2002 National Health Interview Survey (NHIS), which included an extensive set of questions on the use of CAM.

## Methods

The NHIS is an annual survey of the health of the U.S. civilian, non-institutionalized population conducted by the National Center for Health Statistics, Centers for Disease Control and Prevention (CDC). The survey uses a multi-stage clustered sample design [2]. Individual counties or groups of counties comprise primary sampling units. In 2002, non-Hispanic black and Hispanic populations were over-sampled to allow for more accurate national estimates of health for these increasing minority populations.

The survey contains four modules: Household, Family, Sample Child, and Sample Adult. The first two modules collect health and sociodemographic information on each member of all families residing within a sampled household. Within each family, additional information is collected from one randomly selected sample adult aged 18 years or over and from the parent or guardian of one randomly selected sample child under age 18. For the 2002 interview sample, there were 36,161 households consisting of 93,386 persons in 36,831 families. The total household response rate was 89.6%. From the households interviewed, 31,044 adults completed interviews, resulting in an overall sample adult response rate of 74.3%.

This project was approved by the National Center for Health Statistics Institutional Review Board on November 13, 2001. Verbal or written consent was obtained from all survey participants.

### Dependent variable

In 2002, a 10-minute supplement on CAM was added to the NHIS. The NHIS applied the definition of CAM as used by the National Institutes of Health, National Center for Complementary and Alternative Medicine at the time the 2002 survey was designed and fielded [10]. Administered to sample adults, the supplement asked a number of questions about the use of CAM therapies within the past 12 months. CAM use, the dependent variable for this study, was defined as use of any of the following in the past 12 months: acupuncture, Ayurveda, biofeedback, chelation therapy, chiropractic care, energy healing therapy/Reiki, folk medicine, hypnosis, massage, naturopathy, natural herbs, homeopathic treatment, diet-based therapies (specifically, Vegetarian diet, Macrobiotic diet, Atkins diet, Pritikin diet, Ornish diet and Zone diet), high dose or megavitamin therapy, yoga, tai chi, qi gong, and meditation and other relaxation techniques.

### Independent variables

The independent variables in this study are five measures of sample adult health behaviors and risk factors routinely monitored by the CDC [11,12]: (1) leisure-time physical activity (LTPA): regular activity (light or moderate activity performed for at least 30 minutes five or more times per week and/or vigorous activity performed for at least 20 minutes three or more times per week), some activity (less than regular but more than none), and no activity; (2) smoking status: current smoker (smokes everyday or some days), former smoker (smoked at least 100 cigarettes in life, but not currently), and never smoked (smoked fewer than 100 cigarettes in life); (3) drinking status: lifetime abstainer (≤ 12 drinks in lifetime), former drinker (0 drinks in last year, but > 12 drinks in lifetime), infrequent drinker (< one drink per week), light drinker (one to seven drinks per week), moderate drinker (eight to 14 drinks per week), and heavy drinker (15+ drinks per week); (4) body weight status (based on Body Mass Index (BMI)) coded as underweight (<18.5), normal weight (18.5–24.9), overweight (25.0–29.9), and obese (30.0+); and (5) whether or not the respondent received a flu shot in the past 12 months.

### Control variables

A set of 16 variables were employed as controls in the multiple logistic regression models. Nine of these variables related to the respondent's health status, access to conventional care, and use of conventional care. Seven variables represented the sociodemographic characteristics of the sampled adult population. Health status and measures of conventional care access and use included: health compared to 12 months ago, any functional limitation, number of self-reported health conditions, number of visits to a doctor or other health care professional in the past 12 months, whether care was delayed for reasons of cost, or for reasons other than cost, use of prescription or over-the-counter (OTC) medications in the past 12 months, and health insurance status. "Number of self-reported health conditions" is a count variable of approximately 50 chronic and non-chronic conditions found in the NHIS. These include conditions of the cardio-pulmonary, respiratory, musculoskeletal, gastrointestinal, neurological, and endocrine systems. The sociodemographic variables included educational attainment, income (defined in terms of poverty status), gender, age, employment status, race and ethnicity, and region of residence. The complete coding for all variables is presented with the results in Tables 1, 2 and 3.

**Table 1:** Respondent health behaviors and risk factors and associations with CAM use, 2002 NHIS (weighted)

**Characteristic**	**% of Sample**	**% of group using CAM in last 12 months**	**UOR^a^**	**99% CI^b^**	**AOR^c^**	**99% CI^b^**
**Leisure-time physical activity**						
No activity	37.9	23.1	1.00	.	1.00	.
Some activity	30.2	41.0	2.31	2.10–2.54	1.73	1.56–1.92
Regular activity	31.9	46.8	2.92	2.66–3.21	2.38	2.14–2.65
**Smoking status**						
Never smoked	54.9	35.2	1.00	.	1.00	.
Former smoker	22.6	40.6	1.28	1.17–1.40	1.13	1.01–1.26
Current smoker	22.5	34.8	1.02	0.93–1.11	1.03	0.92–1.16
**Drinking status**						
Lifetime abstainer	22.3	24.5	1.00	.	1.00	.
Former drinker	15.2	33.9	1.58	1.40–1.79	1.21	1.04–1.42
Infrequent drinker	31.2	41.6	2.19	1.97–2.44	1.43	1.26–1.61
Light drinker	21.4	42.4	2.27	2.02–2.56	1.59	1.38–1.83
Moderate drinker	6.0	39.7	2.03	1.72–2.40	1.50	1.21–1.85
Heavy drinker	3.4	32.9	1.51	1.21–1.89	1.25	0.97–1.60
**Body mass index**						
Underweight	2.0	33.6	0.83	0.64–1.10	0.84	0.61–1.17
Healthy weight	39.4	37.8	1.00	.	1.00	.
Overweight	35.0	35.9	0.92	0.85–1.00	0.97	0.88–1.06
Obese	23.5	35.0	0.89	0.81–0.98	0.83	0.74–0.93
**Flu shot in past 12 months**						
Yes	28.0	37.2	1.06	0.98–1.15	0.95	0.86–1.05
No	72.0	35.8	1.00	.	1.00	.

^a^Unadjusted odds ratios. Sets of bivariate logistic regressions were performed to determine associations between each independent and control variable and CAM use.

^b^More conservative 99% confidence intervals were used because of the enhanced statistical power generated by the large sample size.

^c ^Adjusted odds ratios. Each variable is adjusted for all other variables in Tables 1, 2, and 3.

**Table 2:** Respondent health status and healthcare access and utilization measures, and associations with CAM use, 2002 NHIS (weighted)

**Characteristic**	**% of Sample**	**% of group using CAM in last 12 months**	**UOR^a^**	**99% CI^b^**	**AOR^c^**	**99% CI^b^**
**Health compared to 12 months ago**						
Worse	8.8	42.7	1.52	1.34–1.72	1.13	0.98–1.31
Same	73.8	32.9	1.00	.	1.00	.
Better	17.4	46.3	1.76	1.60–1.94	1.39	1.25–1.56
**Functional limitation^d^**						
Yes	30.6	43.0	1.53	1.41–1.65	1.30	1.16–1.45
No	69.5	33.1	1.00	.	1.00	.
**Number of health conditions^e^**						
0 conditions	22.5	21.1	1.00	.	1.00	.
1–2 conditions	29.7	32.1	1.77	1.57–1.99	1.55	1.37–1.75
3–5 conditions	25.4	41.8	2.69	2.39–3.03	2.29	1.99–2.64
6+ conditions	22.4	49.0	3.61	3.19–4.08	3.33	2.80–3.97
**Number of doctor visits in past 12 months**						
0 visits	19.0	24.9	1.00	.	1.00	.
1 visit	16.7	30.7	1.33	1.17–1.52	1.05	0.90–1.23
2–3 visits	25.5	36.4	1.72	1.52–1.95	1.21	1.05–1.40
4–9 visits	24.6	40.2	2.03	1.80–2.28	1.30	1.11–1.52
10+ visits	14.2	49.9	3.01	2.62–3.45	1.78	1.49–2.13
**Health insurance status**						
Uninsured	15.5	31.0	1.00	.	1.00	.
Private insurance	70.8	38.9	1.42	1.27–1.58	0.95	0.83–1.10
Public insurance	13.7	27.6	0.85	0.74–0.98	0.83	0.70–0.99
**Delayed conventional care for reasons other than cost^f^**						
Yes	9.0	56.3	2.31	2.05–2.60	1.51	1.32–1.73
No	91.0	43.9	1.00	.	1.00	.
**Delayed conventional care because of cost**						
Yes	9.5	46.6	1.62	1.45–1.81	1.34	1.16–1.54
No	90.5	35.0	1.00	.	1.00	.
**Used prescription medications in past 12 months**						
Yes	67.0	40.0	1.71	1.57–1.87	0.93	0.83–1.04
No	33.0	28.0	1.00	.	1.00	.
**Used over-the-counter medications in past 12 months**						
Yes	78.2	39.1	1.89	1.72–2.08	1.23	1.10–1.37
No	21.8	25.3	1.00	.	1.00	.

^a ^Unadjusted odds ratios. Sets of bivariate logistic regressions were performed to determine associations between each independent and control variable and CAM use.

^b ^More conservative 99% confidence intervals were used because of the enhanced statistical power generated by the large sample size.

^c ^Adjusted odds ratios. Each variable is adjusted for all other variables in Tables 1, 2, and 3.

^d ^"Functional limitation" is defined as any difficulty: walking a quarter of a mile, standing for two hours, stooping/bending/kneeling, climbing 10 steps without resting, sitting for two hours, reaching up over the head, using your fingers to grasp small objects, lifting or carrying a 10-pound item, or pushing/pulling a large object.

^e ^"Number of health conditions" is a count variable of approximately 50 chronic and non-chronic conditions found in the Sample Adult core. These include hypertension, coronary heart disease, angina, heart attack, other heart condition, stroke, emphysema, high cholesterol, poor circulation, irregular heartbeats, congestive heart failure, asthma, ulcer, irritable/inflammatory bowel, thyroid problem, urinary problem, food allergy, allergy to medication, multiple sclerosis, Parkinson's disease, neuropathy, seizures, cancer, diabetes, hay fever, sinusitis, chronic bronchitis, weak or failing kidneys, liver condition, arthritis, diabetic retinopathy, cataracts, glaucoma, macular degeneration, insomnia, fatigue, recurring pain, depression, severe sprain or strains, dental pain, skin problems, joint pain, neck pain, low back pain, facial pain, severe headache or migraine, head or chest cold, and stomach or intestinal illness.

^f ^"Delayed conventional care for reasons other than cost" is coded "yes" if the sample adult: couldn't get through on the telephone, couldn't get an appointment soon enough, had to wait too long to see the doctor, couldn't get to a clinic or doctor's office when open, or didn't have transportation.

**Table 3:** Respondent sociodemographic variables and associations with CAM use, 2002 NHIS (weighted)

**Characteristic**	**% of Sample**	**% of group using CAM in last 12 months**	**UOR^a^**	**99% CI^b^**	**AOR^c^**	**99% CI^b^**
**Educational attainment**						
Less than high school	16.6	20.7	1.00	.	1.00	.
High school graduate/GED	29.9	30.2	1.65	1.45–1.88	1.37	1.19–1.57
Some college/Associate's degree	29.1	40.4	2.59	2.29–2.93	1.85	1.62–2.12
Bachelor's degree	16.3	47.9	3.51	3.06–4.03	2.40	2.05–2.81
Master's, Doctorate, or Professional degree	8.2	52.0	4.14	3.49–4.91	2.79	2.27–3.42
**Poverty status^d^**						
Below poverty level	11.2	27.0	1.00	.	1.00	.
100%<=ratio<200%	17.0	28.9	1.10	0.94–1.28	1.06	0.90–1.25
200% <=ratio<300%	17.9	33.1	1.33	1.15–1.55	1.14	0.96–1.36
300%<=ratio<400%	15.4	36.8	1.57	1.35–1.84	1.18	0.98–1.43
400%<=ratio<500%	12.8	39.3	1.74	1.49–2.05	1.20	0.98–1.47
500%+	25.7	44.8	2.19	1.89–2.54	1.30	1.07–1.56
**Sex**						
Male	48.0	31.5	1.00	.	1.00	.
Female	52.0	40.4	1.48	1.37–1.59	1.55	1.42–1.70
**Age**						
18–44	52.5	36.6	1.62	1.46–1.80	1.73	1.47–2.04
45–64	31.4	40.4	1.90	1.71–2.12	1.64	1.41–1.90
65+	16.1	26.3	1.00	.	1.00	.
**Employment status**						
Not employed	35.9	32.1	1.00	.	1.00	.
Private sector	47.4	36.4	1.21	1.12–1.32	1.07	0.97–1.19
Government	10.2	44.5	1.70	1.49–1.93	1.12	0.95–1.32
Self-employed/family business	6.5	43.5	1.62	1.40–1.89	1.41	1.19–1.66
**Race and ethnicity^e^**						
Hispanic	11.0	27.9	0.63	0.56–0.70	1.09	0.95–1.26
Non-Hispanic white	73.3	38.2	1.00	.	1.00	.
Non-Hispanic black	11.4	28.0	0.63	0.56–0.71	0.90	0.79–1.03
Non-Hispanic American Indian or Alaska Native	0.6	42.4	1.19	0.70–2.03	1.37	0.80–2.37
Asian or Other Pacific Islander	3.7	43.4	1.24	1.01–1.53	1.56	1.19–2.03
**Region of residence**						
Northeast	19.3	37.0	1.31	1.17–1.48	1.12	1.00–1.24
Midwest	24.4	37.9	1.37	1.22–1.53	1.20	1.07–1.35
South	37.0	30.9	1.00	.	1.00	.
West	19.3	43.1	1.70	1.52–1.90	1.58	1.39–1.79

^a ^Unadjusted odds ratios. Sets of bivariate logistic regressions were performed to determine associations between each independent and control variable and CAM use.

^b ^More conservative 99% confidence intervals were used because of the enhanced statistical power generated by the large sample size.

^c ^Adjusted odds ratios. Each variable is adjusted for all other variables in Tables 1, 2, and 3.

^d ^"Poverty status" is based on a multiply-imputed total family income variable.

^e ^Non-Hispanic, multiple race sample adults were dropped from the analysis due to a small sample size (n = 45).

### Statistical analyses

Prevalence estimates for each health behavior/risk factor and control measure are presented for the total sample and for CAM users in Tables 1, 2, and 3. Unadjusted logistic regressions were conducted to identify bivariate associations between independent and control measure and the use of CAM. All control variables were significantly related to CAM use in these bivariate analyses (see Tables 1, 2, and 3) and were therefore retained in the multiple logistic regression analysis. The multivariate analysis will determine whether significant effects of the health behavior and risk factor measures remain after adjusting for the other four health behaviors or risk factors, as well as respondent health status, conventional care access and utilization, and sociodemographic measures. To identify significant relationships with CAM use, more conservative 99% confidence intervals were used in both the unadjusted and adjusted analysis because of the enhanced statistical power generated by the large sample size. For the multiple logistic regression, there was no evidence of collinearity in inspections of tolerance values, condition indices, and variance inflation factors, suggesting properly specified heteroskedastic models.

All estimates, including those of CAM prevalence, were generated using SUDAAN software (version 8.2, Research Triangle Institute, Inc., Research Triangle Park, NC) that accounts for complex sample designs such as that used by the NHIS. To ensure representation of the U.S., civilian, non-institutionalized population age 18 years and over, all estimates were weighted using the NHIS sample adult record weight.

## Results

### Health behaviors/risk factors

In the unadjusted analysis, adults who engaged in regular exercise (46.8%; UOR = 2.92, 99% CI = 2.66–3.21) had almost 3 times the odds of using CAM as persons who did not exercise (23.1%) (Table 1). Former smokers (40.6%; Unadjusted Odds Ratio [UOR] = 1.28, 99% CI = 1.17–1.40) had greater odds of using CAM than did persons who had never smoked (35.2%), whereas there were no differences in CAM use between current smokers and persons who had never smoked. Current or former drinkers were more likely to use CAM than were lifetime abstainers. Among current drinkers, infrequent drinkers (41.6%; UOR = 2.19, 99% CI = 1.97–2.44) had the highest use of CAM and heavy drinkers (32.9%; UOR = 1.51; 99% CI = 1.21–1.89) the lowest. Obese individuals (35.0%; UOR = 0.89, 99% CI = 0.81–0.98) were slightly less likely to use CAM than were individuals of normal weight. No significant associations were observed between CAM use and receipt of the influenza vaccine.

Even after adjusting for the other health behaviors/risk factors, current health status, conventional care access and utilization and sociodemographic measures, physical activity continued to emerge as a strong correlate of CAM use. The associations between CAM use and the other health/behaviors were weaker, with the magnitude of the results attenuated or in some cases eliminated after adjustment (e.g., no difference was seen between CAM use in heavy drinkers and lifetime abstainers).

### Health status, healthcare access and utilization

In the unadjusted analysis, each of the health care status and access and utilization measures had significant associations with the use of CAM: persons whose health had changed in the past 12 months, had a functional limitation, had one or more health conditions, visited a doctor 1 or more times in the past 12 months, delayed conventional care due to cost or for reasons other than cost, or used prescription or over-the-counter medications during the past 12 months had greater odds of using CAM (Table 2). Furthermore, the odds of CAM use increased as both the number of reported health conditions and the number of visits to a doctor increased. In fact, the strongest association with CAM was seen for those individuals reporting 6 or more health conditions (AOR= 3.33, 99% CI= 2.8–3.97). Those who had private health insurance had greater odds of using CAM while those with public insurance had reduced odds of using CAM than those who were uninsured, perhaps reflecting the absence of CAM coverage by Medicare and Medicaid.

Once again, the magnitudes of many observed odds ratios were reduced or even reversed in the adjusted analysis. Adjustment eliminated the relationship between health that was worse 12 months ago and CAM use compared with those whose health status had remained the same. The effect for private health insurance and CAM use disappeared after adjusting for the other variables, as did the relationship between prescription medication use and CAM use.

### Sociodemographic variables

In both the unadjusted and adjusted analysis, sociodemographic variables were significantly associated with CAM use in ways consistent with findings in others studies: persons who use CAM had greater odds of having at least a bachelor's degree and higher income, being female, being younger than 65 years of age, being employed, being a Asian or Other Pacific Islander, and living in the western U.S. (Table 3).

Whereas in the unadjusted data Non-Hispanic black and Hispanic respondents were found to have significantly lower odds of using CAM than were non-Hispanic whites, those differences became insignificant in multivariate analyses. Similarly, after adjusting for the other variables, the relationship between poverty status and CAM use was significant only for the highest income group and the relationship between employment and CAM use was significant only for the self-employed group.

## Discussion

This is one of the first reports derived from a nationally representative U.S. dataset that describes the associations between a range of health behaviors/risk factors and the use of CAM. We found that someone who engages in LTPA, has consumed alcohol in his/her life but is not a current heavy drinker, is a former cigarette smoker, or is not obese is more likely to use CAM. These data begin to address a recommendation by the Institute of Medicine [4] that the associations between CAM use and positive behavioral change be explored. However, it remains to be determined whether the use of CAM and the incorporation of positive health behaviors and/or the reduction of health risk factors occur simultaneously as a result of some life changing event [13] resulting in adoption of a "wellness lifestyle" [14] or whether one precedes, and perhaps elicits, the other. Also to be addressed is whether those who use CAM maintain positive health behaviors and/or curtail negative risk factors over time better than do other individuals.

In our study, leisure-time physical activity had the strongest direct association with CAM use among all the assessed health behaviors. To our knowledge, this observation has not been reported previously. Physical activity has been shown to exert a protective effect on health even in those with generally poor health behaviors [15]. In addition, physical fitness has been associated with younger age, better education, higher income, greater internal (versus external) health locus of control, and higher sense of coherence [16], which is also consistent with CAM use in this report.

Associations between CAM use and smoking status were not observed in earlier surveys of health fair participants [17], individuals attending geriatric clinics [18], and members of managed care and health maintenance organizations [19,20]. Although participants in these surveys appeared to be healthier than our NHIS participants, in our analysis, adjusting for the number of health conditions and physician visits, and the use of pharmaceutical drugs had little effect on the odds ratios for the effect of smoking status on CAM use. Because we performed a cross-sectional analysis, the direction of causality, whether these individuals stopped smoking or used CAM first, cannot be directly assessed. However, several converging lines of evidence suggest the possibility of a time sequence. First, unpublished data from the NHIS suggest that a sizable number of CAM users do so for self-management of addictive behaviors [21]. Second, in our data, CAM was more strongly associated with former smoking than with current smoking. This is consistent with smokers deciding to quit as part of a move to a healthier lifestyle that could involve CAM. If people first used CAM then quit smoking, we would expect more of an association between CAM and current smoking. Longitudinal analyses will be needed to answer this question definitively.

Previous reports have not agreed on whether, and to what extent, alcohol consumption is associated with CAM use, with some finding an inverse association [19], some a positive association [2], and some no significant relationship [17,18,20]. After adjustment for potential confounds, we found CAM use to be highest among those who consumed light to moderate amounts of alcohol. Of note, some research has identified linkages between light to moderate alcohol consumption and a number of positive health behaviors, including regular physical activity, having a healthy weight, not smoking, and getting influenza vaccinations [22,23]. Taken together, these findings suggest that people make clear lifestyle choices that encompass a range of health-related activities, including CAM use.

We identified a set of noteworthy associations between select measures of health status, healthcare access and utilization, and sociodemographic measures and the use of CAM independent of the relationships between respondents' health behaviors and CAM use. Similar to other reports [1-3,13] we found a particularly strong association between use of CAM and a number of factors indicative of poorer health, such as the number of reported health conditions and the number of reported doctor visits. However, CAM use was also associated with improved health status and increased use of self-care indicators such as LTPA. The latter suggests that while CAM use is most likely among those with current chronic health problems, a subset of CAM users may be healthier (or more health-conscious) than those who do not use CAM. The association between CAM use and frequent physician visits could also be interpreted to reflect more active involvement in care. This is consistent with the concept that a significant portion of CAM use is for prevention, health promotion, and wellness, rather than solely treatment of illness [6].

This study has several limitations. First, the variables being investigated were self-reported. The scientific literature suggests that most people tend to under-report negative health behaviors [24]. Hence, the effects of alcohol consumption [25], for example, may have been diminished in this study. Second, flu shots are very different than the other health behavior indicators we used in that flu shots require contact with a health care provider. As such, obtaining a flu shot is influenced by many factors other than an individual's motivation such as access to care or availability of vaccine, potential confounders we could not account for in our analyses. This might have resulted in our underestimating the association between CAM use and obtaining a flu shot seen by others [26]. Third, these data reflect a cross-sectional set of associations. Longitudinal assessments might have identified cohort and secular trends in the associations between health behaviors and CAM use that were not evident in cross-sectional analysis. Fourth, it is possible that additional respondent health behaviors (as well as other unexplored factors) explain more of the observed relationships. However, the five measures employed are important modifiable factors contributing to fatal diseases and morbidity [27], and can be seen as standard measures of a healthy lifestyle [28]. Finally, because our primary focus was to identify factors associated with the use, versus nonuse, of CAM, a dichotomous dependent variable was utilized. By doing so, information on the number and type of CAM therapies used and frequency of their use was lost. It may be that substantial differences exist between heavy and light users of one or more CAM modalities [6]. It has been found that the use of specific types of CAM therapies is associated with specific personality styles [29]. These associations might confound our results if specific personality styles (e.g., "openness" or "control") are also related to adoption of positive health behaviors.

## Conclusion

Our results demonstrate that individuals who exhibit some positive health behaviors are more likely to use CAM independent of their health status, health access, and various sociodemographic factors. Further studies appear warranted to determine the cause and effect relationships underlying these associations, and the effects on health and quality of life of various CAM modalities, alone and in combination with selected, positive health behaviors. Moreover, knowledge of these specific patterns of use may contribute to tailoring health promotion programs, as well as to enhancing communication between patients and health care providers. For health care providers, understanding the motivations behind a patient's use of CAM may assist in the design of an optimal treatment plan [30]. The fact that users of CAM tend to pursue generally healthy lifestyles suggests that these groups may well be open to additional recommendations toward optimizing their health.

## Competing interests

All authors were employees of the federal government during the planning, analysis and writing of this manuscript, and performed their work as part of their official duties. No outside financial support was provided. All authors declare they have no competing interests.

## Authors' contributions

RN conceived and designed the study, interpreted data, and drafted substantial portions of the manuscript. JD participated in survey design, statistical analysis and data interpretation, and drafted substantial portions of the manuscript. BT participated in data acquisition, statistical analysis and data interpretation, and drafted substantial portions of the manuscript. PB participated in data acquisition, statistical analysis and data interpretation, and drafted substantial portions of the manuscript. BS participated in survey design, statistical analysis and data interpretation, and drafted substantial portions of the manuscript. CS participated in statistical analysis and data interpretation, and drafted substantial portions of the manuscript. MB participated in study design and data interpretation, and revised substantial portions of the manuscript. MC participated in study design and data interpretation, and revised substantial portions of the manuscript. MJ participated in survey design and data interpretation, and revised substantial portions of the manuscript. HM participated in study design and data interpretation, and revised substantial portions of the manuscript. KM participated in study design, statistical analysis and data interpretation.

All authors read and approved the final manuscript.

## Pre-publication history

The pre-publication history for this paper can be accessed here:


